# LncRNA SNHG15 contributes to doxorubicin resistance of osteosarcoma cells through targeting the miR-381-3p/GFRA1 axis

**DOI:** 10.1515/biol-2020-0086

**Published:** 2020-12-06

**Authors:** Jinshan Zhang, Dan Rao, Haibo Ma, Defeng Kong, Xiaoming Xu, Hougen Lu

**Affiliations:** School of Medicine, Yangtze University, 434020, Jingzhou, China; Central Hospital of Edong Medical Group, Huangshi City, Hubei Province, 435000, Huangshi, China; The Second Clinical Medical College, Yangtze University, No. 1 Renmin Road, Jingzhou City, Hubei Province, 434020, Jingzhou, China

**Keywords:** osteosarcoma, SNHG15, miR-381-3p, GFRA1, cell proliferation, autophagy, chemoresistance

## Abstract

**Background:**

Osteosarcoma is a common primary malignant bone cancer. Long noncoding RNA small nucleolar RNA host gene 15 (SNHG15) has been reported to play an oncogenic role in many cancers. Nevertheless, the role of SNHG15 in the doxorubicin (DXR) resistance of osteosarcoma cells has not been fully addressed.

**Methods:**

Cell Counting Kit-8 assay was conducted to measure the half-maximal inhibitory concentration value of DXR in osteosarcoma cells. Western blotting was carried out to examine the levels of autophagy-related proteins and GDNF family receptor alpha-1 (GFRA1). Quantitative reverse transcription-polymerase chain reaction was performed to determine the levels of SNHG15, miR-381-3p, and GFRA1. The proliferation of osteosarcoma cells was measured by MTT assay. The binding sites between miR-381-3p and SNHG15 or GFRA1 were predicted by Starbase bioinformatics software, and the interaction was confirmed by dual-luciferase reporter assay. Murine xenograft model was established to validate the function of SNHG15 *in vivo*.

**Results:**

Autophagy inhibitor 3-methyladenine sensitized DXR-resistant osteosarcoma cell lines to DXR. SNHG15 was upregulated in DXR-resistant osteosarcoma tissues and cell lines. SNHG15 knockdown inhibited the proliferation, DXR resistance, and autophagy of osteosarcoma cells. MiR-381-3p was a direct target of SNHG15, and GFRA1 bound to miR-381-3p in osteosarcoma cells. SNHG15 contributed to DXR resistance through the miR-381-3p/GFRA1 axis *in vitro*. SNHG15 depletion contributed to the inhibitory effect of DXR on osteosarcoma tumor growth through the miR-381-3p/GFRA1 axis *in vivo*.

**Conclusions:**

SNHG15 enhanced the DXR resistance of osteosarcoma cells through elevating the autophagy via targeting the miR-381-3p/GFRA1 axis. Restoration of miR-381-3p expression might be an underlying therapeutic strategy to overcome the DXR resistance of osteosarcoma.

## 1 Introduction

Osteosarcoma is the most common malignant bone cancer that mostly arises in childhood and adolescence [1]. The pathogenesis of osteosarcoma is very complex [2,3], and a combination of chemotherapy and surgery increased the survival rate of osteosarcoma patients to about 70% [4,5]. Doxorubicin (DXR) and methotrexate are common chemotherapeutic agents for osteosarcoma treatment. Nevertheless, acquired or intrinsic chemoresistance is an enormous challenge for the effective application of chemotherapeutic agents [6]. Therefore, exploring the mechanism of chemoresistance to DXR is essential for osteosarcoma treatment.

Long noncoding RNAs (lncRNAs) are a class of noncoding RNAs (ncRNAs) with more than 200 nucleotides, and generally they are unable to encode proteins [7]. LncRNAs have been reported to exert their functions by acting as microRNA (miRNA) sponges [8,9,10,11]. LncRNA small nucleolar RNA host gene 15 (SNHG15) was found to be upregulated in a variety of cancers including breast cancer, lung cancer, and hepatocellular carcinoma, and the overexpression of SNHG15 was related to the enhanced proliferation and metastasis abilities of cancer cells [12,13,14]. Liu et al. reported that SNHG15 promoted growth, metastasis, and autophagy of osteosarcoma cells through negatively regulating miR-141 [15]. However, the underlying role of SNHG15 in the chemoresistance of osteosarcoma remains to be determined.

MiRNAs are another class of ncRNAs. MiRNAs can bind to the 3′-untranslated region (3′-UTR) of target messenger RNAs (mRNAs) through their “seed” sequence to reduce the levels of target mRNAs. MiR-381-3p has been reported to play a suppressive role in many malignancies, including colorectal cancer, ovarian cancer, and renal cancer [16,17,18]. Xia et al. claimed that lncRNA CAT104 promoted growth and metastasis while restrained the apoptosis of osteosarcoma cells through sponging miR-381 [19]. However, the underlying signal regulatory network behind miR-381-3p in osteosarcoma is not fully addressed.

GDNF family receptor alpha-1 (GFRA1) has been reported to play an oncogenic role in breast cancer and pancreatic cancer [20,21]. Besides, Kim et al. found that GFRA1 contributed to the chemoresistance of osteosarcoma through accelerating autophagy [22]. Herein, we investigated the role of GFRA1 in DXR-induced chemoresistance of osteosarcoma cells.

In this study, we initially evaluated the role of lncRNA SNHG15 in DXR-induced chemoresistance of osteosarcoma cells, and then the mechanism by which SNHG15 contributed to DXR resistance in osteosarcoma was explored.

## 2 Materials and methods

### 2.1 Clinical samples

Thirty DXR-sensitive and 30 DXR-resistant osteosarcoma samples were obtained from patients in Jingzhou Central Hospital, Second Clinical College of Yangtze University.


**Informed consent:** Informed consent has been obtained from all individuals included in this study.
**Ethical approval:** The research related to human use has been complied with all the relevant national regulations, institutional policies and in accordance with the tenets of the Helsinki Declaration, and has been approved by the Ethic Committee of Jingzhou Central Hospital, Second Clinical College of Yangtze University.

### 2.2 Cell culture

Human osteoblast cell line (hFOB) and osteosarcoma cell lines (SaOS-2, U2OS, HOS, and MG63) were acquired from BeNa Culture Collection (Beijing, China) and were maintained in Dulbecco’s Modified Eagle Medium (Gibco, Carlsbad, CA, USA) supplemented with 10% fetal bovine serum (Gibco), 10% penicillin (100 IU/mL), and 10% streptomycin (100 µg/mL). Cells were incubated in a humidified incubator at 37°C and with 5% CO_2_.

DXR was obtained from Meiji Seika Pharma (Tokyo, Japan). The DXR-resistant osteosarcoma cell lines, U2OS/DXR and MG63/DXR, were established by exposing parental U2OS and MG63 cells to gradually increasing concentrations of DXR [23]. Autophagy inhibitor 3-methyladenine (3-MA) was purchased from Sigma (St. Louis, MO, USA).

### 2.3 Cell Counting Kit-8 (CCK-8) assay

To assess cell viability and the half-maximal inhibitory concentration (IC_50_) values of DXR, CCK-8 assay (Sigma) was used according to the manufacturer’s instructions. The absorbance at 450 nm was measured using a microplate reader.

### 2.4 Western blotting

Osteosarcoma cells were lysed with RIPA lysis solution (Beyotime, Shanghai, China). Proteins extracted from osteosarcoma cells were separated by sodium dodecyl sulfate–polyacrylamide gel electrophoresis and transferred to polyvinylidene fluoride membranes. The membranes were blocked with nonfat milk for 1 h at room temperature and then incubated at 4°C overnight with the following primary antibodies: anti-LC3 (1:5000, ab51520, Abcam), anti-Beclin-1 (1:8000, ab210498, Abcam), anti-p62 (1:5000, ab155686, Abcam), anti-GFRA1 (1:5000, ab84106, Abcam), and anti-glyceraldehyde-3-phosphate dehydrogenase (GAPDH; 1:20000, ab37168, Abcam). Afterward, the membranes were incubated with the horseradish peroxidase-conjugated secondary antibody (1:5000, ab205718, Abcam) for 2 h at room temperature. Protein bands were detected through the enhanced chemiluminescence system (Beyotime).

### 2.5 Quantitative reverse transcription-polymerase chain reaction (qRT-PCR)

RNA samples were isolated from tissues and cells using Trizol reagent (Invitrogen, Carlsbad, CA, USA). For SNHG15 and GFRA1, complementary DNA (cDNA) was synthesized using Bio-Rad iScript kit (Bio-Rad, Hercules, CA, USA). For miR-381-3p, reverse transcription was performed using TaqMan reverse transcription kit (Applied Biosystems, Rotkreuz, Switzerland). qPCR was performed using SYBR Green detection reagent (Cowin Biotech, Beijing, China) and gene-specific primers (GeneCopoeia, Rockville, MD, USA) on the Bio-Rad CFX96 (Bio-Rad). The PCR conditions were as follows: 95°C for 5 min, followed by 39 cycles of 95°C for 30 sec and 60°C for 30 sec. The levels of SNHG15, miR-381-3p, and GFRA1 were calculated by the 2^−ΔΔCt^ method and normalized to U6 or β-actin [24]. The following primers were used: SNHG15 (forward 5′-CAACCATAGCGGTGCAACTGTGC-3′, reverse 5′-GGCTGAACCAAGTTGCAAGTCATG-3′), miR-381-3p (forward 5′-AGTCTATACAAGGGCAAGCTCTC-3′, reverse 5′-ATCCATGACAGATCCCTACCG-3′), GFRA1 (forward 5′-TGTCAGCAGCTGTCTAAAGG-3′, reverse 5′-CTTCTGTGCCTGTAAATTTGCA-3′), U6 (forward 5′-CTCGCTTCGGCAGCACA-3′, reverse 5′-AACGCTTCACGAATTTGCGT-3′), and β-actin (forward 5′-AGCCTCGCCTTTGCCGA-3′, reverse 5′-CTGGTGCCTGGGGCG-3′).

### 2.6 Cell transfection

Small interfering RNA (siRNA) negative control (si-NC), siRNA against SNHG15 (si-SNHG15), siRNA against GFRA1 (si-GFRA1), pcDNA, and SNHG15 overexpression plasmid (pcDNA-SNHG15) were purchased from GenePharma (Shanghai, China). Anti-miRNA-NC (anti-miR-NC), anti-miR-381-3p, miR-NC, and miR-381-3p were synthesized by RiboBio (Guangzhou, China). All transfection experiments were conducted using Lipofectamine 2000 (Invitrogen, Carlsbad, CA, USA).

### 2.7 3-(4,5-Dimethylthiazol-2-yl)-2,5-diphenyltetrazolium bromide (MTT) assay

MTT assay was used to assess cell proliferation. After transfection, the cells were seeded in 96-well plates. Following treatment with 10 µL of MTT (Invitrogen) for 4 h and addition of 200 µL of DMSO (Sigma), the absorbance was measured at 490 nm.

### 2.8 Dual-luciferase reporter assay

Two reporter vectors, namely SNHG15 WT and SNHG15 MUT, containing wild-type or mutant-type binding sites with miR-381-3p were constructed. After co-transfection with miR-NC or miR-381-3p in osteosarcoma cells, the dual-luciferase reporter assay kit (Promega, Madison, WI, USA) was used to determine the luciferase activity. Analogously, the targeted relationship between miR-381-3p and GFRA1 was also verified by the dual-luciferase reporter assay.

### 2.9 Murine xenograft model

Nude mice were obtained from Orient Bio Inc (Seongnam, South Korea). The stable SNHG15-knockdown MG63/DXR cells were built by transfection with short hairpin RNA (shRNA) against SNHG15 (sh-SNHG15; Santa Cruz Biotechnology, Dallas, TX, USA), and MG63/DXR cells stably transfected with sh-NC were used as the control. The mice (n = 5 in each group) were subcutaneously injected with the abovementioned MG63/DXR cells stably transfected with sh-NC or sh-SNHG15. DXR (5.0 mg/kg) and PBS were injected into mice through the tail vein every 2 days since the average tumor size reached about 50 mm^3^. The tumor volume was recorded every 4 days and calculated using the formula: width^2^ × length × 0.5. The mice were sacrificed after 31 days of inoculation, and the tumors were dissected to measure weight and detect the levels of SNHG15, miR-381-3p, and GFRA1.


**Ethical approval:** The research related to animal use has been complied with all the relevant national regulations and institutional policies for the care and use of animals and has been approved by the Animal Research Committee of Jingzhou Central Hospital, Second Clinical College of Yangtze University.

### 2.10 Statistical analysis

All experiments were repeated three times and data were presented as mean ± standard deviation. Statistical significance was analyzed by Student’s *t*-test or one-way analysis of variance followed by Tukey’s test. The correlation between the expression of miR-381-3p and SNHG15 or GFRA1 in DXR-resistant osteosarcoma tissues was analyzed through Spearman’s correlation coefficient. *P* < 0.05 was considered statistically significant.

## 3 Results

### 3.1 DXR resistance is related to autophagy in osteosarcoma cells

**Figure 1:**
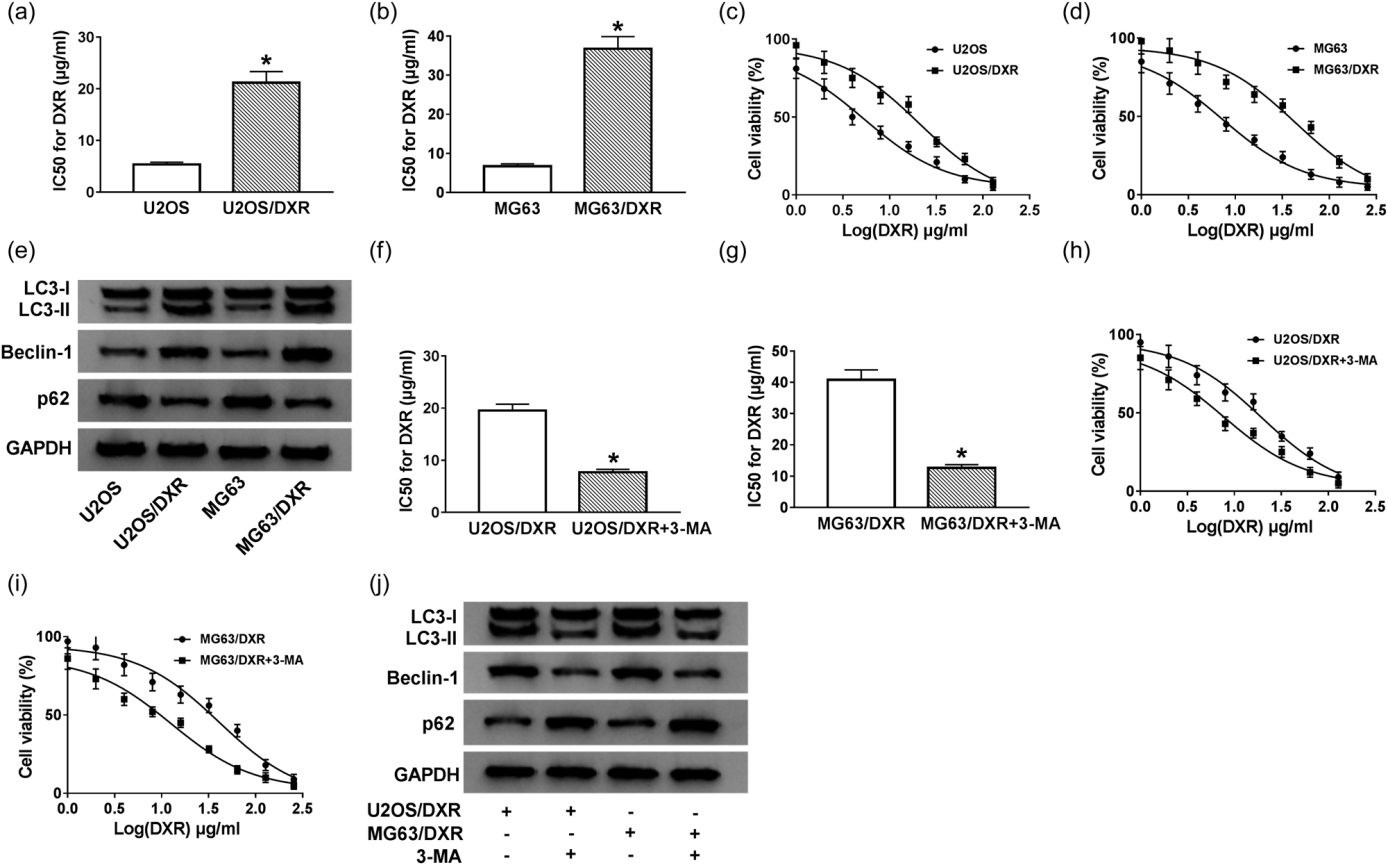
DXR resistance is related to autophagy in osteosarcoma cells. (a and b) The IC_50_ value of DXR was measured in U2OS and MG63 cells and their matching DXR-resistant subclones (U2OS/DXR and MG63/DXR) by CCK-8 assay. (c and d) Cell viability of U2OS, U2OS/DXR, MG63, and MG63/DXR cells treated with different concentrations of DXR was determined by CCK-8 assay. (e) Western blotting was conducted to examine autophagy-related proteins (LC3, Beclin-1, and p62) in U2OS, U2OS/DXR, MG63, and MG63/DXR cells. (f and g) CCK-8 assay was performed to detect the IC_50_ value of DXR in U2OS/DXR, U2OS/DXR treated with autophagy inhibitor 3-MA, MG63/DXR, and MG63/DXR cells treated with 3-MA. (h and i) U2OS/DXR, U2OS/DXR pretreated with 3-MA, MG63/DXR, and MG63/DXR cells pretreated with 3-MA were treated with different concentrations of DXR, and CCK-8 assay was carried out to measure the viability of these cells. (j) Western blotting was conducted to examine autophagy-related proteins in U2OS/DXR, U2OS/DXR treated with 3-MA, MG63/DXR, and MG63/DXR cells treated with 3-MA. *P < 0.05.

**Figure A1:**
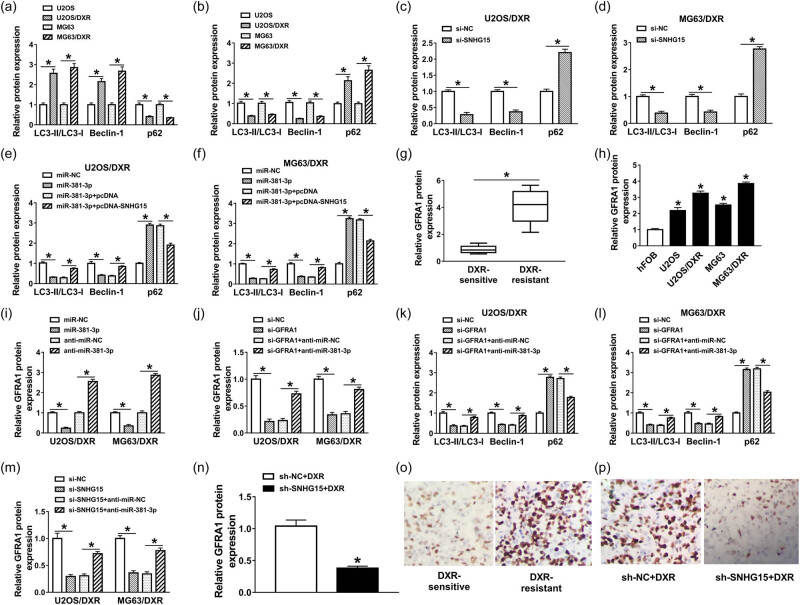
Quantitative data from Western blots. (a) Quantitative data from Figure 1e. (b) Quantitative data from Figure 1j. (c and d) Quantitative data from Figure 3i and j. (e and f) Quantitative data from Figure 5i and j. (g and h) Quantitative data from Figure 6e and g. (i) Quantitative data from Figure 6k. (j) Quantitative data from Figure 7c. (k and l) Quantitative data from Figure 7j and k. (m) Quantitative data from Figure 8d. (n) Quantitative data from Figure 9g. (o) The expression of GFRA1 in DXR-resistant and -sensitive osteosarcoma tissues was examined by IHC. (p) The expression of GFRA1 in tumor tissues from nude mice in the sh-SNHG15 + DXR group and the sh-NC + DXR group was analyzed by IHC. *P < 0.05.

To clarify the drug-resistance mechanism of osteosarcoma cells, two DXR-resistant osteosarcoma cell lines (U2OS/DXR and MG63/DXR) were established. The IC_50_ value was measured for DXR in the parental osteosarcoma cells (U2OS and MG63) and their matching DXR-resistant subclones (U2OS/DXR and MG63/DXR) to assess whether the DXR-resistant osteosarcoma cell lines were successfully established. As shown in Figure 1a–d, the IC_50_ value of DXR was prominently elevated in DXR-resistant subclones compared with that in their parental cells. The expression of LC3-II/LC3-I and Beclin-1 was also elevated, while p62 was downregulated in U2OS/DXR and MG63/DXR cells compared with that in their parental cells (Figures 1e and A1a), suggesting that the DXR resistance of osteosarcoma cells might be related to the autophagy. The 3-MA is a common autophagy inhibitor. To validate our hypothesis, the IC_50_ value was measured in U2OS/DXR and MG63/DXR cells treated with control or 3-MA. As shown in Figures 1j and A1b, autophagy was inhibited in 3-MA-treated U2OS/DXR and MG63/DXR cells. As shown in Figure 1f–i, the addition of 3-MA elevated the DXR sensitivity of two DXR-resistant osteosarcoma cell lines. These findings suggested that DXR resistance was associated with autophagy in osteosarcoma cells.

### 3.2 The expression of SNHG15 is elevated in DXR-resistant osteosarcoma tissues and cells

**Figure 2:**
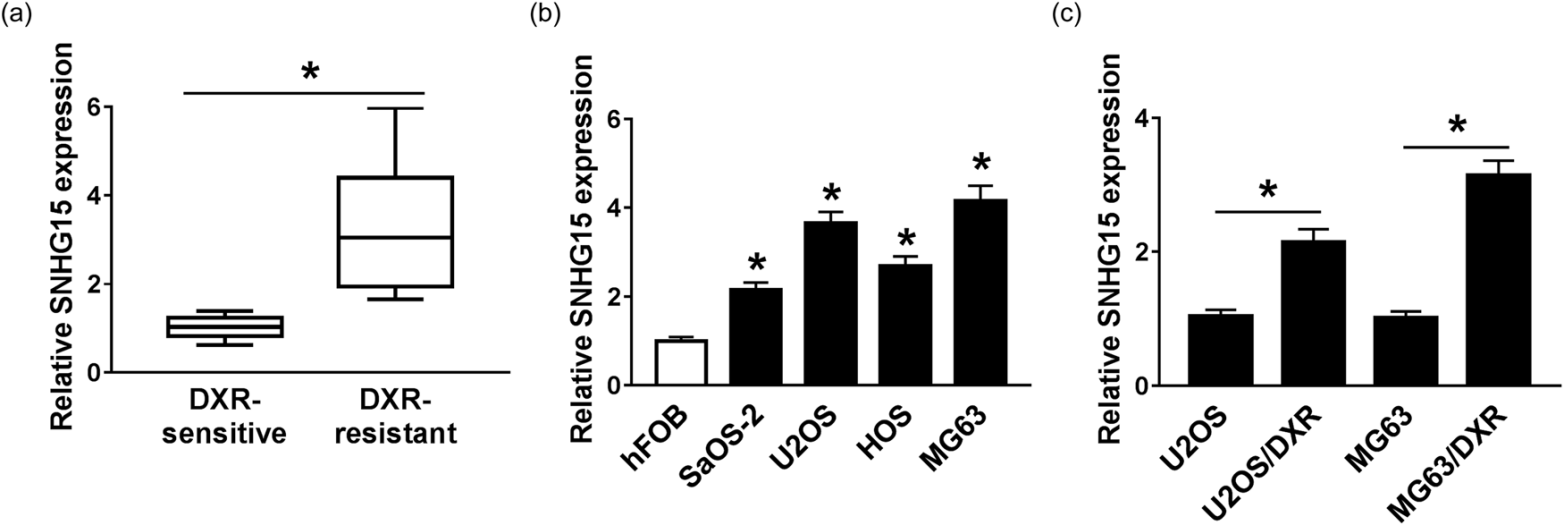
SNHG15 is elevated in DXR-resistant osteosarcoma tissues and cells. (a) The expression of SNHG15 was determined in DXR-resistant and -sensitive osteosarcoma tissues by qRT-PCR. (b) The level of SNHG15 was measured in human osteoblast cells hFOB and osteosarcoma cells (SaOS-2, U2OS, HOS, and MG63) by qRT-PCR. (c) The expression of SNHG15 was detected in U2OS and MG63 cells and their matching DXR-resistant subclones (U2OS/DXR and MG63/DXR) by qRT-PCR. *P < 0.05.

The expression of SNHG15 was upregulated in DXR-resistant osteosarcoma tissues (*n* = 30) compared with that in DXR-sensitive osteosarcoma tissues (*n* = 30) (Figure 2a), suggesting that it might play a crucial role in DXR resistance of osteosarcoma cells. As indicated in Figure 2b, the enrichment of SNHG15 was enhanced in osteosarcoma cells compared with that in human osteoblast cells hFOB. Besides, the expression of SNHG15 was further upregulated in DXR-resistant osteosarcoma cells compared with that in their parental cells (Figure 2c).

### 3.3 SNHG15 promotes proliferation, autophagy, and chemoresistance in osteosarcoma cells

**Figure 3:**
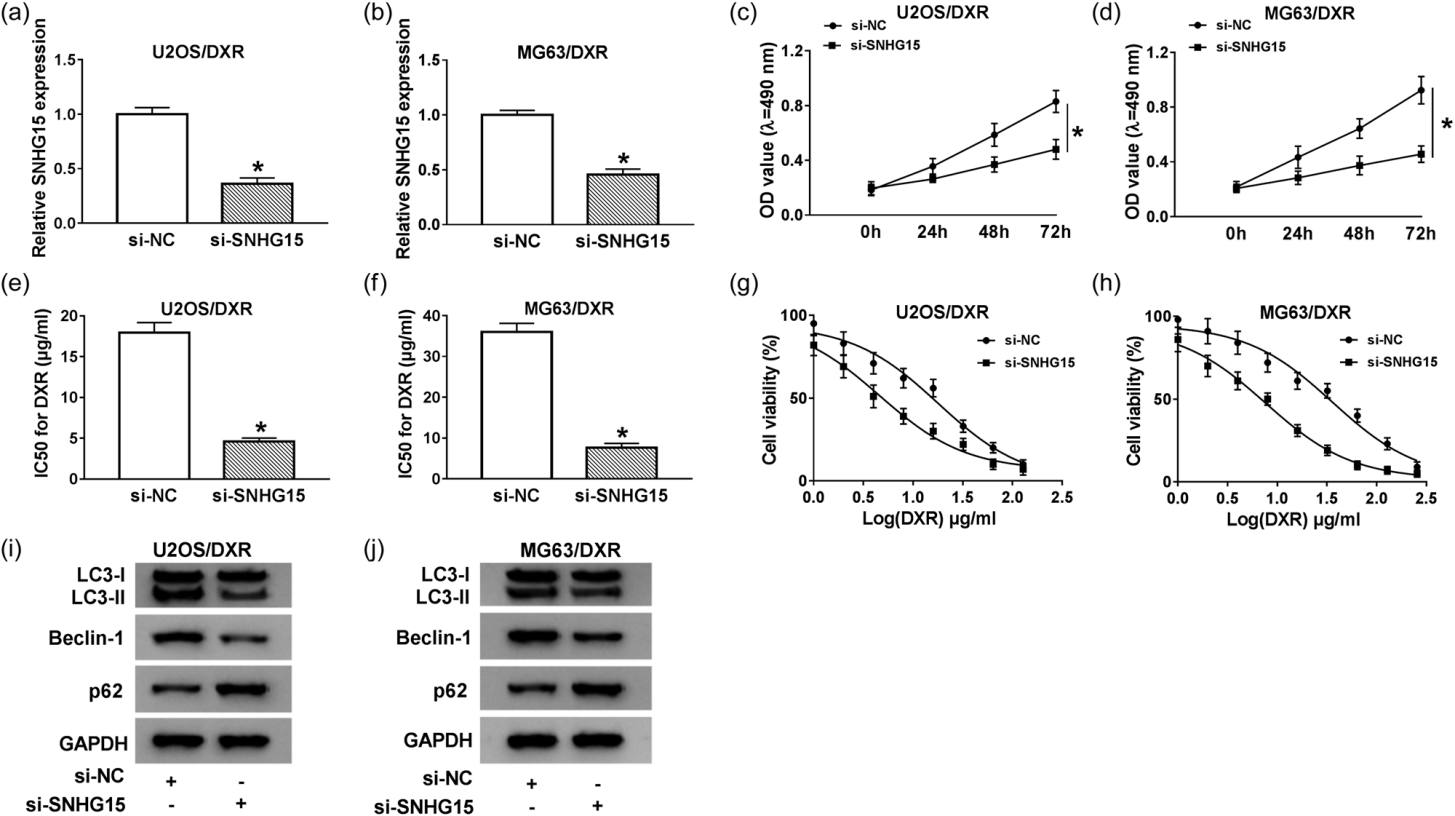
SNHG15 promotes proliferation, autophagy, and chemoresistance in osteosarcoma cells. (a and b) The knockdown efficiency of si-SNHG15 was assessed in U2OS/DXR and MG63/DXR cells by qRT-PCR. (c and d) Cell proliferation of U2OS/DXR and MG63/DXR cells transfected with si-NC or si-SNHG15 was detected by MTT assay. (e and f) The IC_50_ value of DXR was measured in U2OS/DXR and MG63/DXR cells transfected with si-NC or si-SNHG15 by CCK-8 assay. (g and h) U2OS/DXR and MG63/DXR cells transfected with si-NC or si-SNHG15 were treated with different concentrations of DXR, and CCK-8 assay was carried out to measure the viability of these cells. (i and j) Western blotting was conducted to examine autophagy-related proteins in si-NC or si-SNHG15-transfected U2OS/DXR and MG63/DXR cells. *P < 0.05.

As mentioned above, DXR resistance of osteosarcoma cells was related to autophagy. Therefore, the hypothesis was proposed that SNHG15 contributed to DXR resistance of osteosarcoma cells through accelerating autophagy. We assessed the knockdown efficiency of si-SNHG15 in U2OS/DXR and MG63/DXR cells. As shown in Figure 3a and b, the level of SNHG15 notably decreased in the si-SNHG15 group compared with that in the si-NC group. Proliferation was restrained in U2OS/DXR and MG63/DXR cells transfected with si-SNHG15 compared with that in the si-NC group (Figure 3c and d). Meanwhile, SNHG15 knockdown enhanced DXR sensitivity of two DXR-resistant osteosarcoma cells (Figure 3e–h). Autophagy was restrained in si-SNHG15-transfected DXR-resistant osteosarcoma cells compared with that in the si-NC group (Figures 3i, j and A1c, d). Taken together, SNHG15 interference upregulated the DXR sensitivity of osteosarcoma cells via inhibiting the autophagy of osteosarcoma cells.

### 3.4 MiR-381-3p is a direct target of SNHG15 in osteosarcoma cells

**Figure 4:**
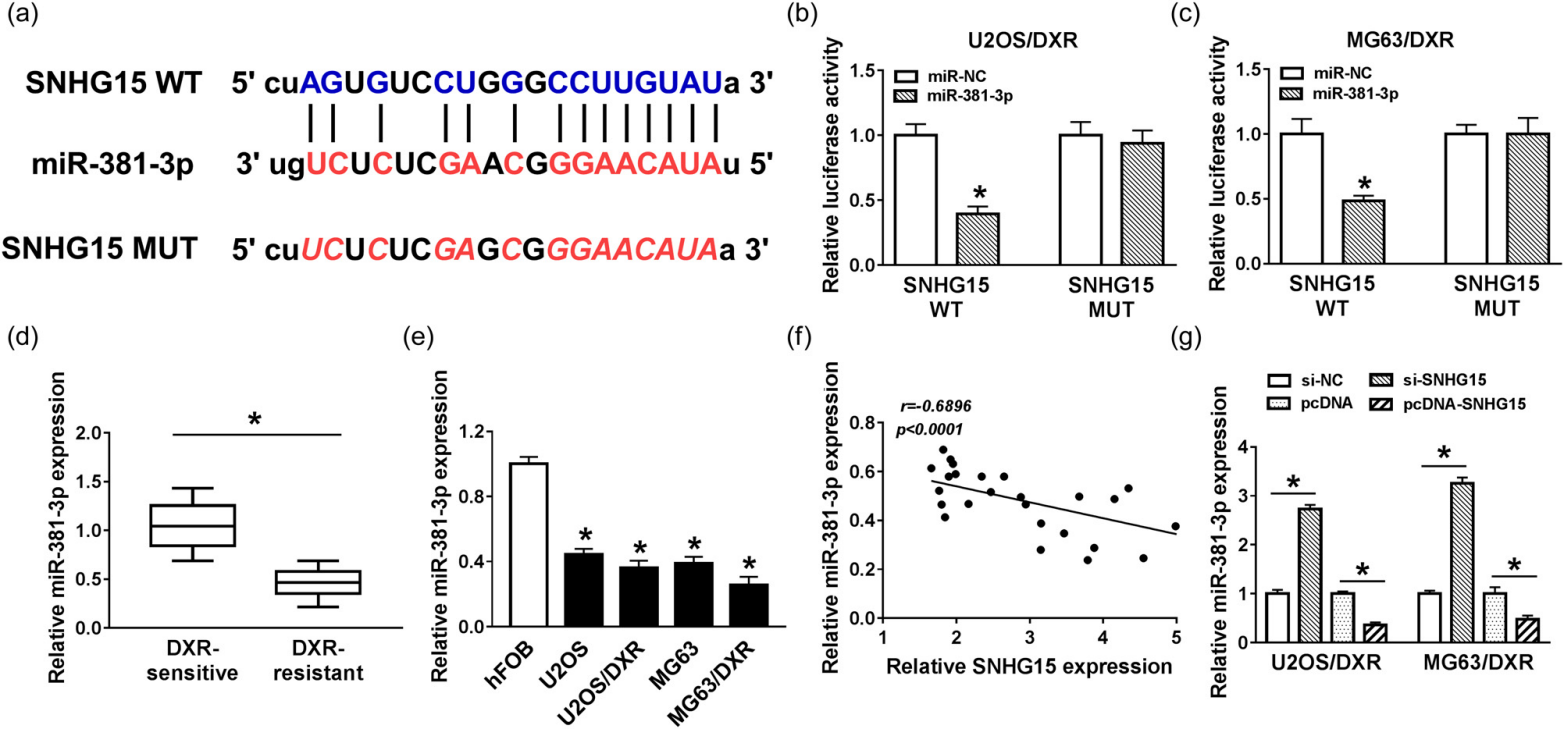
MiR-381-3p is a direct target of SNHG15 in osteosarcoma cells. (a) The binding sites between SNHG15 and miR-381-3p were predicted by Starbase software. (b and c) Dual-luciferase reporter assay was conducted to verify the interaction between SNHG15 and miR-381-3p in U2OS/DXR and MG63/DXR cells. (d) The expression of miR-381-3p was determined in DXR-resistant and -sensitive osteosarcoma tissues by qRT-PCR. (e) The level of miR-381-3p was determined in hFOB, U2OS, U2OS/DXR, MG63, and MG63/DXR cells by qRT-PCR. (f) Correlation analysis was performed to analyze the relationship between miR-381-3p and SNHG15 in DXR-resistant osteosarcoma tissues. (g) The expression of miR-381-3p in U2OS/DXR and MG63/DXR cells transfected with si-NC, si-SNHG15, pcDNA, or pcDNA-SNHG15 was measured by qRT-PCR. *P < 0.05.

To illustrate the mechanism by which SNHG15 elevated the DXR resistance of osteosarcoma cells, the downstream genes of SNHG15 were investigated. As shown in Figure 4a, miR-381-3p was predicted as a target of SNHG15 by Starbase software. The luciferase activity was dramatically reduced in miR-381-3p and SNHG15 WT co-transfected groups compared with that in miR-381-3p and SNHG15 MUT co-transfected groups, suggesting that miR-381-3p was a direct target of SNHG15 in osteosarcoma cells (Figure 4b and c). To elucidate the role of miR-381-3p in osteosarcoma, we first measured the expression of miR-381-3p in DXR-sensitive and -resistant osteosarcoma tissues and cells. As shown in Figure 4d and e, the level of miR-381-3p notably decreased in DXR-resistant osteosarcoma tissues and cells. The expression of miR-381-3p was negatively correlated with the level of SNHG15 in DXR-resistant osteosarcoma tissues (Figure 4f). To further clarify the modulatory relationship between SNHG15 and miR-381-3p in osteosarcoma cells, we transfected si-NC, si-SNHG15, pcDNA, or pcDNA-SNHG15 into U2OS/DXR and MG63/DXR cells. As indicated in Figure 4g, the level of miR-381-3p elevated with the transfection of si-SNHG15, and the accumulation of SNHG15 reduced the expression of miR-381-3p. Collectively, miR-381-3p was a direct target of SNHG15 in osteosarcoma cells, and it was negatively modulated by SNHG15.

### 3.5 SNHG15 contributes to DXR resistance of osteosarcoma cells through sponging miR-381-3p to promote autophagy

**Figure 5:**
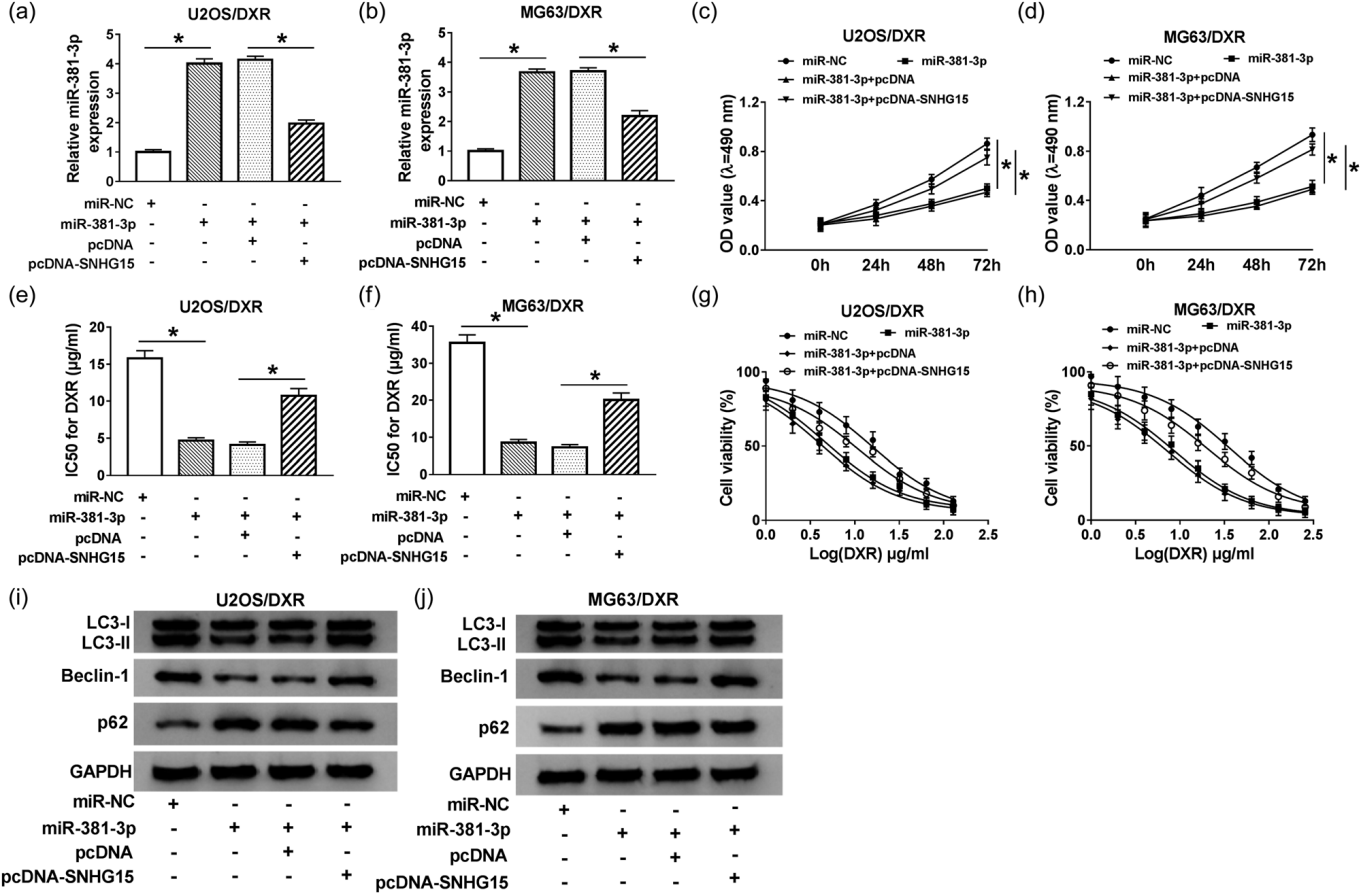
SNHG15 contributes to DXR resistance of osteosarcoma cells through sponging miR-381-3p. U2OS/DXR and MG63/DXR cells were transfected with miR-NC, miR-381-3p, miR-381-3p + pcDNA, or miR-381-3p + pcDNA-SNHG15. (a and b) The expression of miR-381-3p was examined by qRT-PCR. (c and d) Cell proliferation was detected by MTT assay. (e and f) The IC_50_ value of DXR was determined by CCK-8 assay. (g and h) The abovementioned osteosarcoma cells were treated with different concentrations of DXR, and CCK-8 assay was carried out to measure cell viability. (i and j) The autophagy-related proteins were detected by Western blotting. *P < 0.05.

We measured the level of miR-381-3p in U2OS/DXR and MG63/DXR cells transfected with miR-NC, miR-381-3p, miR-381-3p + pcDNA, or miR-381-3p + pcDNA-SNHG15. As shown in Figure 5a and b, the expression of miR-381-3p elevated with the transfection of miR-381-3p in U2OS/DXR and MG63/DXR cells, and it decreased with the addition of pcDNA-SNHG15. The accumulation of SNHG15 reversed the inhibitory effect of miR-381-3p overexpression on the proliferation of osteosarcoma cells (Figure 5c and d). Besides, the overexpression of SNHG15 also counteracted the suppressive effect of miR-381-3p accumulation on the DXR resistance of U2OS/DXR and MG63/DXR cells (Figure 5e–h). Apart from this, the autophagy of osteosarcoma cells was restrained with miR-381-3p accumulation, while the co-transfection of miR-381-3p and pcDNA-SNHG15 recovered the autophagy of osteosarcoma cells (Figures 5i, j and A1e, f). These results revealed that SNHG15 contributed to the DXR resistance of osteosarcoma cells via inversely modulating miR-381-3p to promote autophagy.

### 3.6 GFRA1 binds to miR-381-3p in osteosarcoma cells

**Figure 6:**
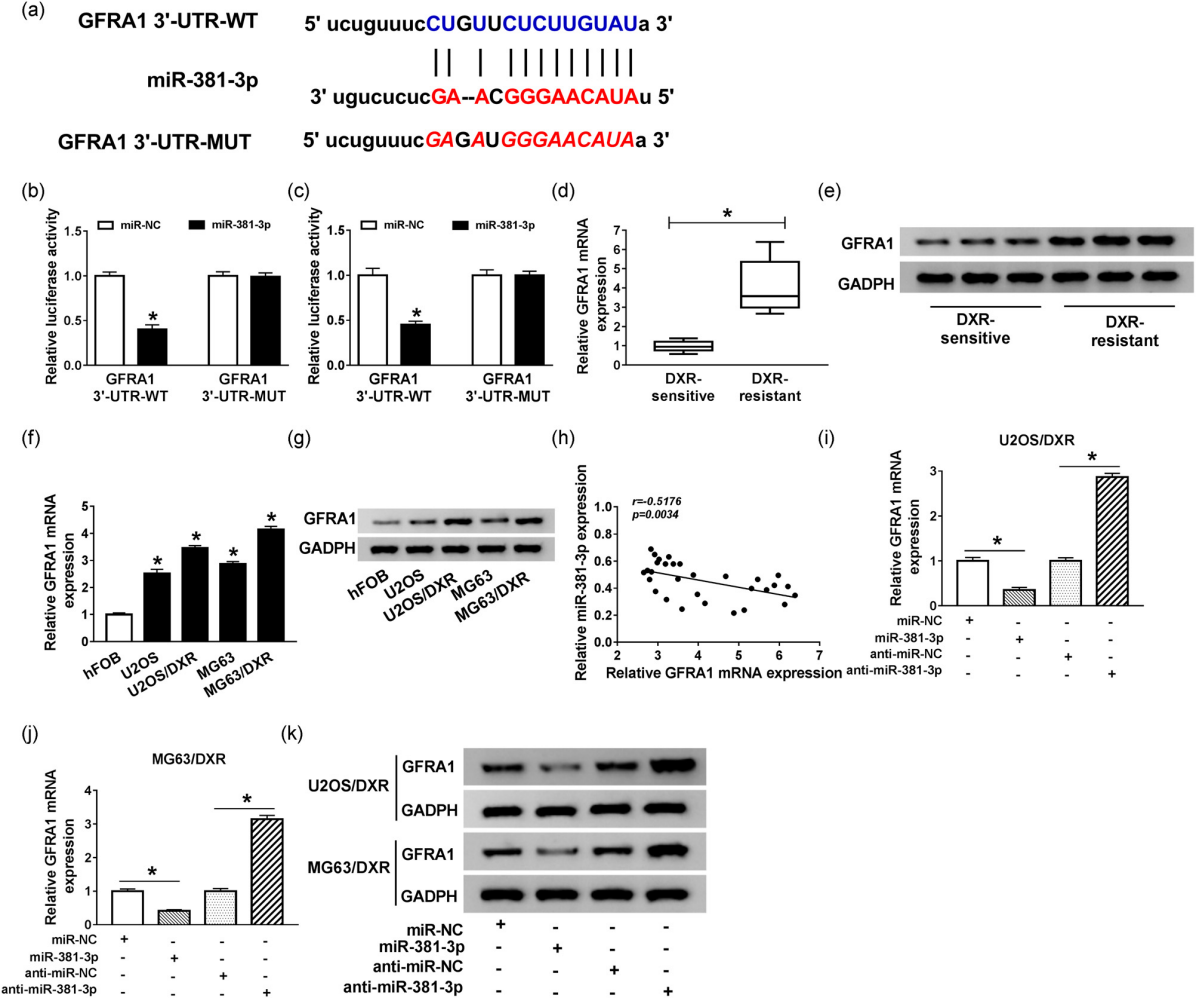
GFRA1 binds to miR-381-3p in osteosarcoma cells. (a) GFRA1 was predicted to be a target of miR-381-3p based on Starbase software. (b) Dual-luciferase reporter assay was conducted to verify the interaction between miR-381-3p and GFRA1 in U2OS/DXR cells. (c) The direct interaction between miR-381-3p and GFRA1 in MG63/DXR cells was verified by dual-luciferase reporter assay. (d and e) The expression of GFRA1 mRNA and protein was measured in DXR-resistant and -sensitive osteosarcoma tissues by qRT-PCR and Western blotting. (f and g) The mRNA and protein levels of GFRA1 were detected in hFOB, U2OS, U2OS/DXR, MG63, and MG63/DXR cells by qRT-PCR and Western blotting. (h) The linear relationship between the expression of miR-381-3p and GFRA1 in DXR-resistant osteosarcoma tissues was assessed by Spearman’s correlation coefficient. (i–k) The expression of GFRA1 mRNA and protein was measured in U2OS/DXR and MG63/DXR cells transfected with miR-NC, miR-381-3p, anti-miR-NC, or anti-miR-381-3p by qRT-PCR and Western blotting. *P < 0.05.

GFRA1 was predicted as a target of miR-381-3p by Starbase bioinformatics software (Figure 6a). Dual-luciferase reporter assay showed that the luciferase activity conspicuously decreased in U2OS/DXR and MG63/DXR cells co-transfected with miR-381-3p and GFRA1 3′-UTR-WT, whereas it remained unchanged in the abovementioned cells co-transfected with miR-381-3p and GFRA1 3′-UTR-MUT, demonstrating that miR-381-3p directly bound to GFRA1 in osteosarcoma cells (Figure 6b and c). To elucidate the biological significance of GFRA1 in osteosarcoma, the expression of GFRA1 was initially measured in DXR-sensitive and -resistant osteosarcoma tissues and cells. As shown in Figures 6d–g and A1g, h, the mRNA and protein expression of GFRA1 were markedly upregulated in DXR-resistant osteosarcoma tissues and cells. The results of immunohistochemistry (IHC) also revealed that GFRA1 was notably upregulated in DXR-resistant osteosarcoma tissues compared with DXR-sensitive osteosarcoma tissues (Figure A1o). Correlation analysis showed a negative relationship between the levels of miR-381-3p and GFRA1 in DXR-resistant osteosarcoma tissues (Figure 6h). Besides, the expression of GFRA1 mRNA and protein was negatively regulated by miR-381-3p in osteosarcoma cells (Figures 6i–k and A1i). Taken together, GFRA1 was a direct target of miR-381-3p, and it was inversely modulated by miR-381-3p in osteosarcoma cells.

### 3.7 MiR-381-3p increases the DXR sensitivity of osteosarcoma cells by negatively regulating GFRA1

**Figure 7:**
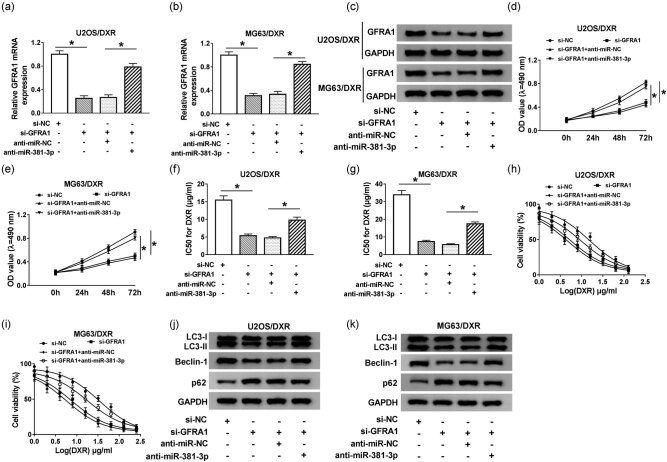
MiR-381-3p increases the DXR sensitivity of osteosarcoma cells by negatively regulating GFRA1. U2OS/DXR and MG63/DXR cells were transfected with si-NC, si-GFRA1, si-GFRA1 + anti-miR-NC, or si-GFRA1 + anti-miR-381-3p. (a–c) The expression of GFRA1 mRNA and protein was detected by qRT-PCR and Western blotting. (d and e) Cell proliferation was detected by MTT assay. (f and g) The IC_50_ value of DXR was measured by CCK-8 assay. (h and i) The abovementioned osteosarcoma cells were treated with different concentrations of DXR, and CCK-8 assay was carried out to measure cell viability. (j and k) The autophagy-related proteins were detected by Western blotting. *P < 0.05.

To illustrate whether miR-381-3p regulated DXR sensitivity of osteosarcoma cells through GFRA1, U2OS/DXR and MG63/DXR cells were transfected with si-NC, si-GFRA1, si-GFRA1 + anti-miR-NC, or si-GFRA1 + anti-miR-381-3p. GFRA1 interference decreased the mRNA and protein expression of GFRA1 in osteosarcoma cells, while the addition of anti-miR-381-3p recovered the expression of GFRA1 mRNA and protein (Figures 7a–c and A1j). As shown in Figure 7d–i, miR-381-3p depletion abolished the inhibitory effects of GFRA1 interference on the proliferation and chemoresistance of osteosarcoma cells. Meanwhile, the autophagy was restrained with GFRA1 depletion in osteosarcoma cells, and the co-transfection of anti-miR-381-3p and si-GFRA1 recovered the autophagy function of U2OS/DXR and MG63/DXR cells (Figures 7j, k and A1k, l). These findings suggested that miR-381-3p enhanced the DXR sensitivity of osteosarcoma cells by downregulating GFRA1.

### 3.8 GFRA1 is regulated by the SNHG15/miR-381-3p axis in osteosarcoma cells

**Figure 8:**
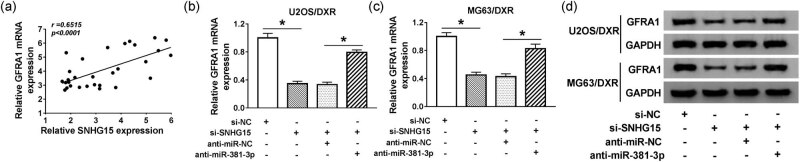
GFRA1 is regulated by the SNHG15/miR-381-3p axis in osteosarcoma cells. (a) The linear relationship between the expression of SNHG15 and GFRA1 in DXR-resistant osteosarcoma tissues was evaluated by Spearman’s correlation coefficient. (b–d) The expression of GFRA1 mRNA and protein was measured in U2OS/DXR and MG63/DXR cells transfected with si-NC, si-SNHG15, si-SNHG15 + anti-miR-NC, or si-SNHG15 + anti-miR-381-3p by qRT-PCR and Western blotting. *P < 0.05.

To further illustrate the regulatory relationship among SNHG15, miR-381-3p, and GFRA1 in osteosarcoma cells, we first analyzed the linear relationship between the expression of SNHG15 and GFRA1 in DXR-resistant osteosarcoma tissues. As shown in Figure 8a, a positive relationship was observed between GFRA1 and SNHG15 in DXR-resistant osteosarcoma tissues. Subsequently, we examined the mRNA and protein levels of GFRA1 in U2OS/DXR and MG63/DXR cells transfected with si-NC, si-SNHG15, si-SNHG15 + anti-miR-NC, or si-SNHG15 + anti-miR-381-3p. The expression of GFRA1 mRNA and protein notably reduced with SNHG15 depletion in the two osteosarcoma cells, and miR-381-3p interference abated the inhibitory effect of SNHG15 interference on the mRNA and protein expression of GFRA1 (Figures 8b–d and A1m). Collectively, GFRA1 was modulated by SNHG15/miR-381-3p signaling in osteosarcoma cells.

### 3.9 SNHG15 knockdown enhances the DXR sensitivity of osteosarcoma cells through the miR-381-3p/GFRA1 axis *in vivo*

**Figure 9:**
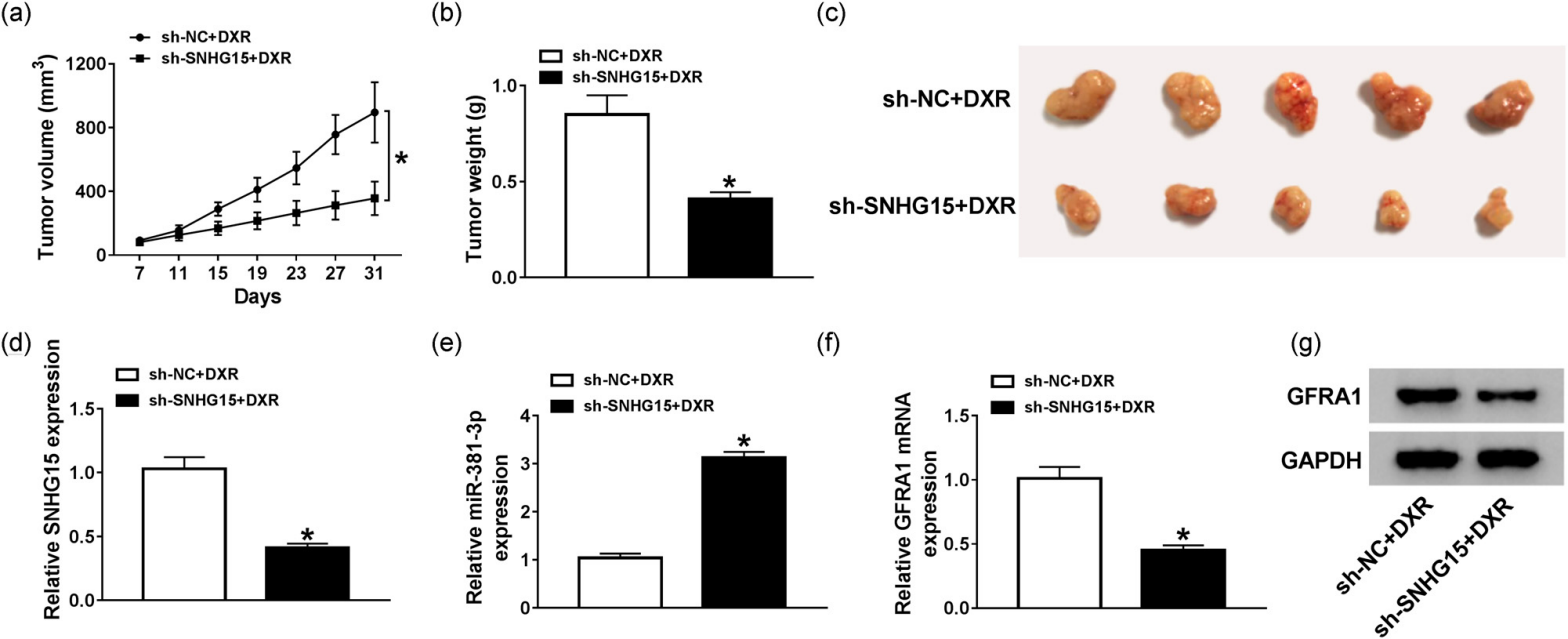
SNHG15 knockdown enhances the DXR sensitivity of osteosarcoma cells through the miR-381-3p/GFRA1 axis *in vivo*. (a) Tumor volume was measured every 4 days after 1 week of inoculation. (b) Tumors were weighed after 31 days of inoculation. (c) The representative images of tumors in the sh-NC + DXR group and the sh-SNHG15 + DXR group. (d and e) The expression of SNHG15 and miR-381-3p was detected in resected tumor tissues by qRT-PCR. (f and g) The mRNA and protein levels of GFRA1 were determined in resected tumor tissues by qRT-PCR and Western blotting *P < 0.05.

We established murine xenograft model using MG63/DXR cells stably transfected with sh-NC or sh-SNHG15. As shown in Figure 9a and b, the tumor volume and weight were lesser in the sh-SNHG15 + DXR group than that in the sh-NC + DXR group, suggesting that SNHG15 knockdown contributed to the inhibitory effect of DXR on the tumor growth *in vivo*. The representative images of dissected tumors in the sh-NC + DXR group and the sh-SNHG15 + DXR group are shown in Figure 9c. The level of SNHG15 decreased, and the expression of miR-381-3p elevated in the sh-SNHG15 + DXR group (Figure 9d and e). Besides, the mRNA and protein levels of GFRA1 reduced in the sh-SNHG15 + DXR group compared with that in the sh-NC + DXR group (Figures 9f, g and A1n). The results of IHC showed that GFRA1 protein significantly reduced in tissue samples from the sh-SNHG15 + DXR group compared with that in the sh-NC + DXR group (Figure A1p), which was consistent with the results of Figure 9g. Taken together, SNHG15 contributed to DXR resistance of osteosarcoma cells through the miR-381-3p/GFRA1 axis *in vivo*.

## 4 Discussion

Autophagy contributes to chemoresistance and the viability of cancer cells under diverse stress conditions [25,26,27]. Herein, it was found that autophagy was notably promoted in DXR-resistant osteosarcoma subclones (U2OS/DXR and MG63/DXR), and the autophagy inhibitor 3-MA sensitized DXR-resistant subclones to DXR, suggesting that the chemoresistance to DXR of osteosarcoma cells was associated with autophagy. SNHG15 has been reported to be upregulated in many cancers [12,13,14]. For instance, Chen et al. reported that SNHG15 was elevated in gastric cancer tissues compared with that in the corresponding normal tissues, and the level of SNHG15 was positively correlated with tumor staging and lymph node metastasis [28]. We found that lncRNA SNHG15 was conspicuously elevated in DXR-resistant osteosarcoma tissues and cells. To illustrate the role of SNHG15 in the chemoresistance of osteosarcoma cells, loss-of-function experiments were conducted. The knockdown of SNHG15 suppressed the proliferation, DXR resistance, and autophagy of osteosarcoma cells. These findings revealed that SNHG15 promoted the DXR resistance of osteosarcoma cells through promoting autophagy.

To uncover the underlying signaling pathway by which SNHG15 contributed to the DXR resistance of osteosarcoma cells, the targets of SNHG15 were explored. MiR-381-3p was predicted as a target of SNHG15 by Starbase software, and this interaction was confirmed by the dual-luciferase reporter assay. Huang et al. demonstrated that miR-381 sensitized non-small cell lung cancer cells to cisplatin via nuclear factor-κB [29]. Mi et al. found that miR-381 elevated the sensitivity of breast cancer cells to DXR through the FYN/MAPK axis [30]. Yi et al. demonstrated that miR-381 sensitized breast cancer cells to cisplatin [31]. Consistent with the above findings, we found that miR-381-3p enhanced DXR sensitivity of osteosarcoma cells, and SNHG15 accumulation reversed the inhibitory effects of miR-381-3p overexpression on the proliferation, DXR resistance, and autophagy of osteosarcoma cells, demonstrating that SNHG15 contributed to the DXR resistance of osteosarcoma cells through sponging miR-381-3p. GFRA1 was identified as a functional target of miR-381-3p by the dual-luciferase reporter assay. GFRA1 depletion inhibited the proliferation, DXR resistance, and autophagy of osteosarcoma cells, and the addition of anti-miR-381-3p counteracted the suppressive effects caused by GFRA1 depletion, suggesting that GFRA1 was a downstream functional target gene of miR-381-3p in osteosarcoma cells.

We further clarified the modulatory relationship among SNHG15, miR-381-3p, and GFRA1 in osteosarcoma cells. SNHG15 interference downregulated the level of GFRA1, and the co-transfection of anti-miR-381-3p and si-SNHG15 recovered the expression of GFRA1 in osteosarcoma cells. These results showed that GFRA1 was modulated by the SNHG15/miR-381-3p axis in osteosarcoma cells.

The murine xenograft model was built using MG63/DXR cells stably transfected with sh-SNHG15 or sh-NC to assess the effect of SNHG15 interference on DXR sensitivity of osteosarcoma cells *in vivo*. The results revealed that SNHG15 contributed to DXR resistance via the miR-381-3p/GFRA1 axis *in vivo*.

In summary, SNHG15 contributed to DXR resistance of osteosarcoma cells through promoting autophagy via the miR-381-3p/GFRA1 axis *in vivo* and *in vitro*. The SNHG15/miR-381-3p/GFRA1 axis might provide new insights into developing an effective strategy to overcome the DXR resistance of osteosarcoma cells.
